# The global prevalence of turnover intention among general practitioners: a systematic review and meta-analysis

**DOI:** 10.1186/s12875-020-01309-4

**Published:** 2020-11-30

**Authors:** Xing Shen, Heng Jiang, Hongbin Xu, Jun Ye, Chuanzhu Lv, Zuxun Lu, Yong Gan

**Affiliations:** 1 Department of Social Medicine and Health Management, School of Public Health, Tongji Medical College, Huazhong University of Science and Technology, No. 13 Hangkong Road, Wuhan, 430030 Hubei China; 2 Centre for Alcohol Policy Research, School of Psychology and Public Health, La Trobe University, Melbourne, Victoria Australia; 3 Melbourne School of Population and Global Health, University of Melbourne, Melbourne, Victoria Australia; 4 Department of Public Management, School of Public Health and Management, Wenzhou Medical University, Wenzhou, Zhejiang China; 5 Department of Emergency, Hainan Clinical Research Center for Acute and Critical Diseases, The Second Affiliated Hospital of Hainan Medical University, No.3 Xueyuan Road, Longhua Zone, Haikou, 571199 China; 6 Emergency and Trauma College, Hainan Medical University, Haikou, Hainan China; 7 Research Unit of Island Emergency Medicine, Chinese Academy of Medical Sciences (No. 2019RU013) , Hainan Medical University, Haikou, Hainan China; 8 Key Laboratory of Emergency and Trauma of Ministry of Education, Hainan Medical University, Haikou, Hainan China

**Keywords:** Turnover intention, General practitioners, Risk factors, Meta-analysis

## Abstract

**Background:**

General practitioners (GPs) are the foundation of any primary healthcare system. Their quality and quantity are directly associated with the effectiveness and quality of the health services of a nation. GPs’ shortage and turnover have become an important issue in developed and developing countries.

An accurate estimate of turnover intention prevalence among GPs would have important health policy implications, but the overall prevalence is unknown. We aimed to summarize the global prevalence of turnover intention and associated factors among GPs.

**Methods:**

We systematically reviewed the PubMed, Embase, Web of Science and China National Knowledge Infrastructure (CNKI) databases from their inception up to May 2020, as well as the reference lists of all included studies. We included observational studies that reported data on turnover intention or their prevalence rate among GPs could be calculated based on the information provided. The prevalence rate of the turnover intentions was estimated using a random-effects meta-analysis. The heterogeneity was evaluated using *I*^*2*^ statistic. Differences by study level characteristics were estimated via subgroup analysis and meta-regression.

**Results:**

A total of 25 cross-sectional studies were included (a total of 27,285 participants). The prevalence of turnover intention was 0.47 (95% CI: 0.39–0.55). Those having a lower level of salary (OR = 1.38, 95% CI: 1.13–1.63) and job satisfaction (OR = 1.35, 95% CI: 1.12–1.70) or having lower level of morale (OR = 2.68, 95% CI: 1.56–3.80) had a higher intention. In contrast, GPs with a lower level of professional title had a lower turnover intention (OR = 0.81, 95% CI: 0.65–0.98).

**Conclusions:**

In this systematic review, approximately half of the GPs had the intention to leave their current posts worldwide. The factors associated with turnover intention were higher professional title, lower income level, lower job satisfaction and lower morale.

**Supplementary Information:**

The online version contains supplementary material available at 10.1186/s12875-020-01309-4.

## Background

General practitioners (GPs) are medical professionals affiliated with primary medical service institutions who provide primary care and health management services [1, 2]. GPs mainly respond to common diseases and general emergencies [3]. GPs often efficiently provide care, quickly treat ailments, and somewhat justify referrals and hospitalizations for difficult cases [4]. In 2019, there were 44,570 fulltime GPs in the United Kingdom, which was an increase of 2.7% since 2018 [5]. GPs are important contributors to effective primary care serving [6, 7] as gatekeepers by promoting full primary care coverage [8].

Some recent previous studies have found that GPs were highly motivated to leave their affiliations [9]. Even in the United Kingdom, where GPs are relatively established, recruitment has been difficult; many job vacancies exist while many GPs consider early retirement or seek employment with lighter clinical burdens [10]. Frequent turnover of GPs is likely to influence a system’s ability to deliver primary care services, which, in turn, might undermine efforts to ensure public health.

Turnover is the actual leaving behavior essential to human resource management of a workforce [11]. Intention to leave (turnover intention) is about an individual’s vision of a possible leaving and it often is studied as a proxy for actual turnover [12]. Studies on turnover intention offer indirect insights into leaving behavior, and some studies have found that GPs’ intentions to leave general practice related to their actual turnover [13]. Intention to leave might reflect low morale, absenteeism, poor performance, and understanding it might help organizations find ways to prevent or reduce actual turnover. Therefore, investigating turnover intention among GPs is important because understanding it might help to target policies and interventions that reduce turnover and improve the quality of primary care.

The determinants of turnover intention often are categorized as extrinsic or intrinsic factors. Extrinsic factors relate to external indicators, such as professional title, salary, and personal development, whereas intrinsic factors tend to be individuals’ work-related psychological factors, such as morale and job satisfaction. Extrinsic factors are strong determinants of turnover intention, and improving them might strengthen employment stability. Attention to intrinsic factors mostly seems to focus on inspiring work-related enthusiasm. Previous studies on GPs’ turnover intention reported that low income [14], poor working conditions [15], low job satisfaction [16, 17], high work-related stress, and frequent workplace violence [18, 19] were associated with turnover [20, 21]. These factors also might influence medical students to avoid the GP specialty, which might influence the supply of and demand for GPs [22, 23].

Many previous studies have investigated the prevalence of and factors related to turnover intention among GPs. A previous review revealed factors that might influence GPs’ actual turnover, but the research generally lacks comprehensive data analysis [24]. The current study investigated the status and risk factors of turnover intention among GPs worldwide. Based on the conclusions drawn by the studies in the meta-analysis, we speculated on the influences of various factors. The findings provide an important reference for healthcare management and for human resource departments as a basis for developing policies and interventions to reduce or eliminate the factors that contribute to turnover intention among GPs. The ultimate goal of this study was to help strengthen and stabilize the global GP workforce.

## Methods

### Search strategy and selection criteria

A systematic review and meta-analysis following the PRISMA and MOOSE guidelines was performed to assess the status and factors related to GPs turnover intention [25, 26]. We conducted a comprehensive search of PubMed, Embase, Web of Science, and the China National Knowledge Infrastructure (CNKI) databases from inception through May 2020 for pertinent studies on turnover intention among GPs. The search terms were “general practitioners or GPs or turnover intention or demission or turnover or health worker or retain.” Only articles published in English or Chinese were considered. Moreover, we manually scrutinized the reference lists of the retrieved articles for additional relevant articles.

All of the studies retrieved by the comprehensive search were screened by title or abstract and then by a full-text assessment. We began selecting articles by screening the titles and abstracts of the articles retrieved from the database search. When relevance could not be determined by screening titles and abstracts, the full text was reviewed. Then, the full texts of all the articles assessed as possibly relevant were reviewed. .

Two researcher (X.S. and H.X.) chose potentially relevant articles based on the titles or abstracts, and two other researchers (Y.G. and H. J.) reviewed those articles to build the final dataset based on the following inclusion criteria: (1) observational study design (cross-sectional, case-control and cohort studies), (2) sample defined as GPs aged 18 years or older, and (3) the article reported the turnover intention rate of GPs or provided sufficient information for it to be calculated. We excluded reviews, essays, letters, and commentaries. When multiple articles reporting the same study sample were identified, the article with the most complete information on results or that reported on the largest number of cases was chosen for the dataset.

### Data extraction

Two of the researchers separately performed the data extraction, compared their results, and resolved inconsistencies by reaching consensus through discussion. We used a predefined and standardized data extraction form developed specifically for this study to collect information from the dataset. These data were author names, years of publication, study sites, sample sizes, and, regarding the subjects, mean ages, genders, prevalence rates, and factors identified by the studies as associated with turnover intention. In cases where the information was not in articles, it was requested from the articles’ corresponding authors.

### Quality assessment

To assess the quality of the studies reported by the articles in the dataset, we used an 11-item index recommended by the Agency for Healthcare Research and Quality. Three items assessed the quality of the studies’ sample selection methodology (e.g., inclusion/exclusion criteria), five items assessed the quality of the variables (e.g., data source, reliability/validity assessment statistics), and three items assessed the quality of the analytical methods (e.g., management of missing data, extent of confounding variables). The response options were yes, no, or unsure. The scoring system assigned one point to articles that indicated the study included the item (yes) and zero points when information was missing (no) or we were unable to determine whether it had been considered (unsure). The scores ranged from zero to 11 points, with higher scores indicating higher quality. Supplement Table 1 reports the distribution of scores. Two of the researchers reviewed the quality ratings of the articles’ studies, and inter-rater reliability on titles, abstracts, and full-text screenings was determined using Cohen’s κ. Quality assessment was also conducted using the Quality in Prognosis Studies (QUIPS) tool [27]. Each study was assessed for risk of bias through six domains: study participation, study attrition, prognostic factor measurement, confounding measurement and account, outcome measurement, analysis and reporting. Supplement Table 3 reported the distribution of scores. For each domain, two review authors (Y.G. and Z.L.) independently assigned a rating of low, moderate, or high risk of bias. Discrepancies were resolved through discussion.

### Data analysis

Turnover intention rate was calculated in the meta-analysis using a random effects model. The extent of statistical heterogeneity across the articles was estimated by *I*^*2*^, and the values at 25%, 50%, and 75% were the cut-off points of low, moderate, and high heterogeneity, respectively [28].

To identify the factors associated with GPs’ turnover intention, the odds ratios (ORs) and 95% confidence intervals (CIs) of predictive factors were pooled and examined in a random effects model.

Sensitivity analyses were used to investigate the sources of heterogeneity. Variations in the turnover intention rates were tested by study period, rural/urban study site, employment setting, and data collection method. Study quality and group differences were tested to investigate heterogeneity across groups. Group analyses by gender, age, work tenure, professional title, salary, individual development, job satisfaction, and morale were performed to examine the influences of associated factors. All the group differences were tested in meta-regression analyses [29]. Publication bias was assessed using the Egger’s regression test, and the cut-off value to determine publication bias was *P* <  0.10. All statistical analyses were performed in STATA 12.0. Except as otherwise specified, all tests of significance were two-tailed and the cut-off value of statistical significance was *P* <  0.05.

## Results

### Study selection

**Fig. 1:**
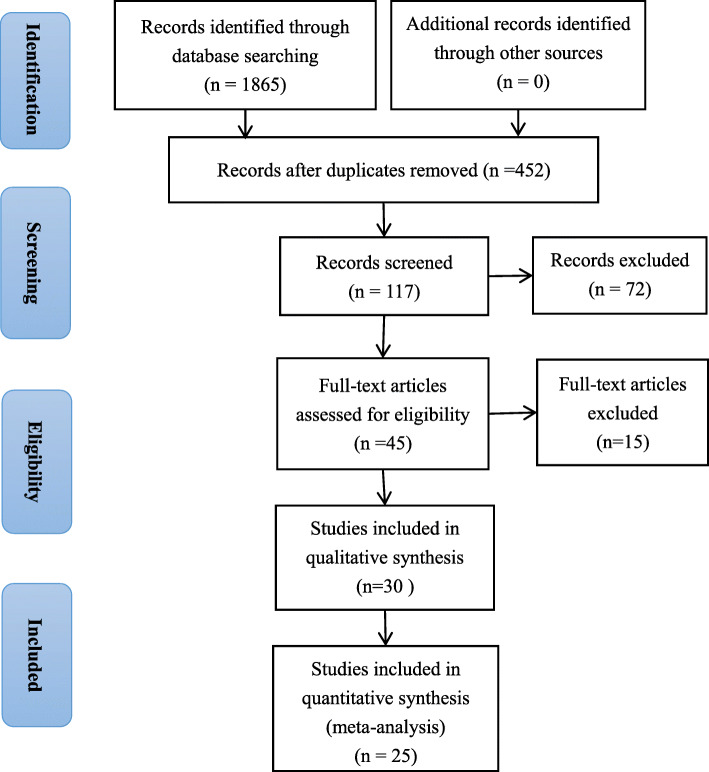
Flow chart of identification of relevant observational studies

Figure 1 illustrates the study selection, identification, and inclusion process using the Preferred Reporting Items for Systematic Reviews and Meta-Analyses flow chart. First, 1865 articles were retrieved from the PubMed, Embase, Web of Science, and CNKI databases. After the initial screening of titles and abstracts, 45 articles remained for full-text assessment. After the detailed full-text evaluation, 30 studies comprised the analytical sample. Of them, three articles’ data reports were insufficient and two articles reported on the same study, resulting in 25 articles published between 1988 and 2019 in the quantitative synthesis [30–54].

### Article and study characteristics

The main characteristics of the 25 articles in the dataset are shown in Table 1. Nine were conducted in Asia, eight were set in Europe, and eight were set in Australia/New Zealand. All of the articles reported results on men and women. The sample sizes ranged from 10 to 11,500 (median = 281, interquartile range = 187–1150), and the total number of cases was 27,285. Observer agreement (κ) was 0.93, indicating excellent agreement between raters for article inclusion determination. Overall, the reported studies’ quality was moderate; quality assessment scores were six or higher on 13 articles, with an average score of 5.6, on the 0–11-point scale (Supplementary Table 1).

**Table 1:** Characteristics of studies included in the meta-analysis

Author	Year	Country	Survey method	No. of participants	Sex	Age at baseline, years
Smith et al.	1988	UK	Questionnaire	488	M/F	NA
Montalto et al.	1994	Australia	Questionnaire	51	M/F	NA
Gardiner et al.	2001	Australia	Questionnaire	212	M/F	Mean age = 40
MacIsaac et al.	2001	Australia	Questionnaire and interview	10	M/F	NA
Joyce et al.	2003	Australia	Questionnaire and interview	15	M/F	30^−^ – 50^+^
Chambers et al.	2004	Scotland	Questionnaire	348	M/F	NA
Gardiner et al.	2005	Australia	Questionnaire and interview	187	M/F	NA
Comb ED.	2008	New Zealand	Questionnaire	1000	M/F	NA
Heponiemi et al.	2012	Finnish	Questionnaire	1705	M/F	Mean age = 50.6
Sun et al.	2013	China	Questionnaire	1150	M/F	Mean age = 29.5
Gardiner et al.	2013	Australia	Questionnaire	202	M/F	NA
Zou et al.	2015	China	Questionnaire	163	M/F	Mean age = 37.7
Dale et al.	2015	UK	Questionnaire and interview	1192	M/F	NA
Matthew et al.	2015	Australia	Questionnaire	1214	M/F	40^−^ – 55^+^
Chang et al.	2016	China	Questionnaire	215	M/F	Mean age = 39.6
Yu et al.	2016	China	Questionnaire	258	M/F	35^−^ – 45^+^
Fletcher et al.	2016	UK	Questionnaire	2177	M/F	Mean age = 48
Iacobucci et al.	2016	UK	Questionnaire	11,500	M/F	NA
Mari et al.	2017	Sweden	Questionnaire	281	M/F	45^−^ – 55^+^
Chen et al.	2017	China	Questionnaire	190	M/F	NA
Fan et al.	2017	China	Questionnaire	85	M/F	Mean age = 39.42
Gan et al.	2018	China	Questionnaire	870	M/F	Mean age = 38.7
Sansom et al.	2018	UK	Questionnaire and interview	41	M/F	NA
Ouweilin et al.	2018	China	Questionnaire	1432	M/F	Mean age = 35.81
Gan et al.	2019	China	Questionnaire	3236	M/F	Mean age = 37.4

*Abbreviation*s: *F* female, *M* male, *NA* not available

### Prevalence of turnover intention among GPs

**Fig. 2:**
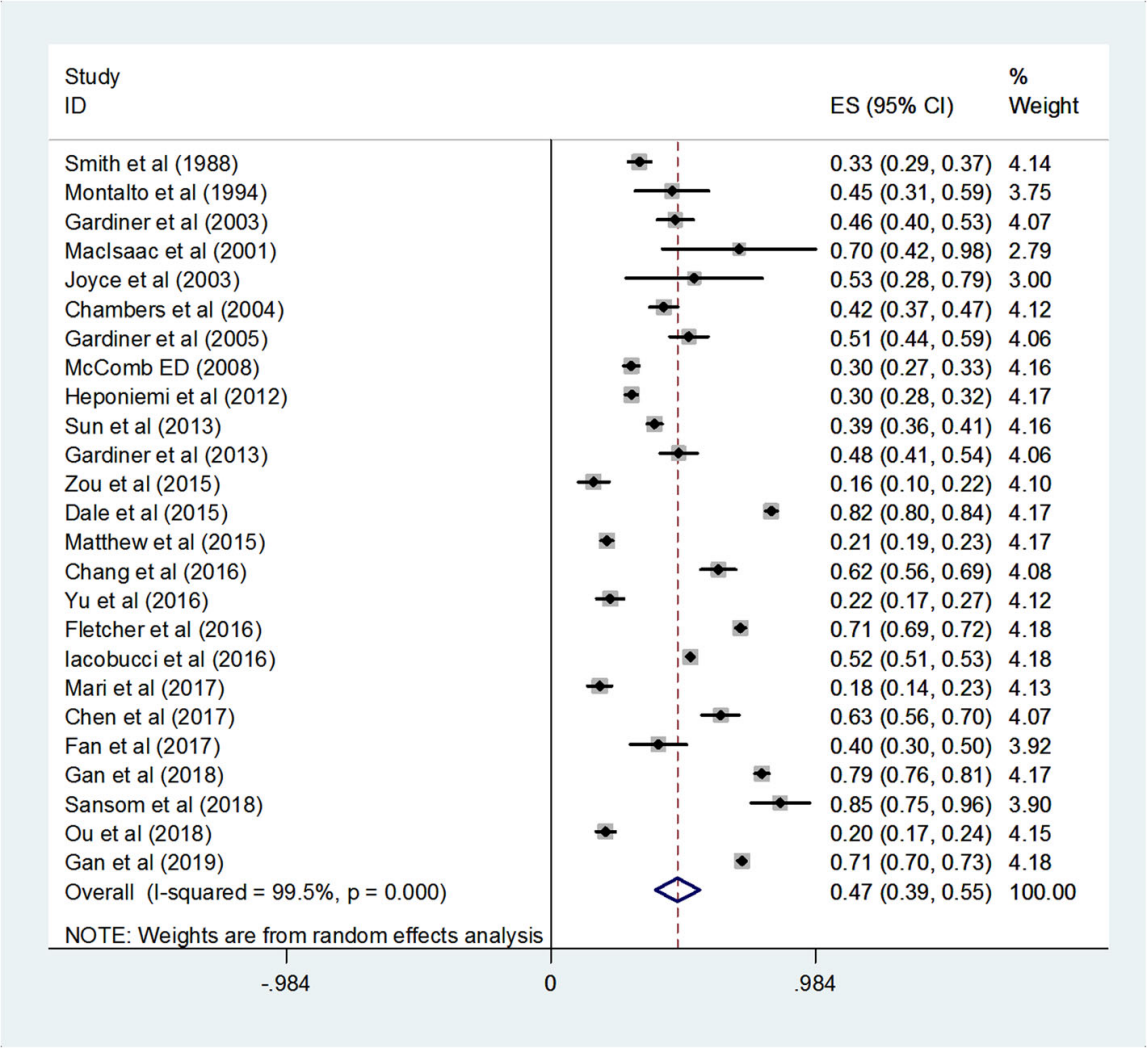
Pooled random effects prevalence rate and 95% Confidence Interval

The pooled turnover intention rate was 0.47 (95% CI = 0.39–0.55), indicating that about 47% of the GPs had a moderate turnover intention. The heterogeneity was *I*^*2*^ = 99.5% (Fig. 2).

### Subgroup analysis

There were no significant differences in turnover intention rates among study site, publication date, and survey method. Groups comparisons found that turnover intention rate data obtained from questionnaires plus interviews (0.69, 95% CI = 0.52–0.86) had much higher rates than those whose data were obtained by questionnaire alone (0.42, 95% CI = 0.34–0.51) (Table 2). Variability was studied after adding survey method variables, and the results of meta-regression showed that survey method could explain 43.77% variability [55].

**Table 2:** Subgroups analyses of prevalence rate of turnover intention among general practitioners

	No. of reports	Prevalence rate (%)	Lower Limit (LL)	Upper Limit (UL)	***I***^***2***^ ***(%)***	***P*** for heterogeneity	***P*** value* between groups
**Primary analysis**	25	0.47	0.39	0.55	99.50	< 0.001	
**Subgroups analyses**							
**Study period**							
2010–2019	17	0.48	0.38	0.58	99.60	< 0.001	0.51
2000–2009	6	0.45	0.36	0.55	91.10	< 0.001	
1988–2000	2	0.37	0.26	0.49	63.80	0.0970	
**Study location**							
Asia	9	0.46	0.29	0.62	99.50	< 0.001	0.664
Australia/New Zealand	8	0.43	0.33	0.52	95.50	< 0.001	
Europe	8	0.51	0.38	0.65	99.60	< 0.001	
**Practice settings**							
Urban community health center	7	0.41	0.22	0.60	99.50	< 0.001	0.95
Rural community health center	8	0.48	0.34	0.49	97.20	< 0.001	
Primary care setting	10	0.47	0.39	0.55	99.60	< 0.001	
**Survey method**							
Questionnaire	20	0.42	0.34	0.51	99.50	< 0.001	0.008
Questionnaire and interview	5	0.69	0.52	0.86	94.30	< 0.001	

*P values for meta-regression

### Factors associated with turnover intention

Four articles reported the odds ratios (OR) and 95% Confidence Intervals (CIs) of associated factors [37, 38, 44, 48]. The analysis of group differences found that the major factors were professional title, salary, personal development, limited opportunities for personal development, job satisfaction, and morale (Table 3). Specifically, GPs with low-level professional titles (OR = 0.81, 95% CI = 0.65–0.98), relatively low salaries (OR = 1.38, 95% CI = 0.65–0.98), limited opportunities for personal development (OR = 1.61, 95% CI = 0.42–1.81), low job satisfaction (OR = 1.35, 95% CI = 1.12–1.70), and low morale (OR = 2.68,95% CI = 1.56–3.80) were more likely than their counterparts to indicate turnover intention.

**Table 3:** Meta-analysis of risk factors associated with turnover intention among general practitioners

Associated factors	Studies (n)	OR	Lower Limit (LL)	Upper Limit (UL)	***I***^***2***^ (%)	***P*** for heterogeneity	Tau-square
Female (ref: Male)	2	1.04	0.79	1.29	0.00	0.81	0.00
Age (ref:≥55 years)	3	1.06	1.02	1.10	91.40	< 0.001	0.09
Work tenures (ref:≥20 years)	2	0.95	0.93	0.98	81	0.02	0.06
Lower professional title (ref: Senior title)	2	0.81	0.65	0.98	0.00	0.93	0.00
Salary (ref:≥¥5000)	2	1.38	1.13	1.63	0.00	0.47	0.00
Individual development (ref: Sufficient opportunities)	2	1.61	0.42	1.81	0.00	0.74	0.00
Job satisfaction (ref: High job satisfaction)	2	1.35	1.12	1.70	89.40	< 0.001	0.11
Morale (ref: High morale)	2	2.68	1.56	3.80	44	0.18	0.69

*Abbreviation*: *OR* odds ratio

### Sensitivity analyses

Sensitivity analyses were performed to explore potential sources of between-study heterogeneity. The pooled turnover intention rate was not materially changed in the leave-one-out analyses by omitting one study in turn. After excluding this study that included far more participants than any other study (*n* = 11,500) [40], the pooled rate did not alter (prevalence rate = 0.51, 95% CI: 0.40–0.59, *I*^*2*^ = 98.7%). In addition, after excluding the study that included fewer participants than any other study (n<100) [32, 35, 51–53], an obvious difference was found (prevalence rate = 0.44, 95% CI: 0.35–0.56, *I*^*2*^ = 76.3%). Therefore, included small sample studies were mainly source of heterogeneity.

### Publication bias

**Fig. 3:**
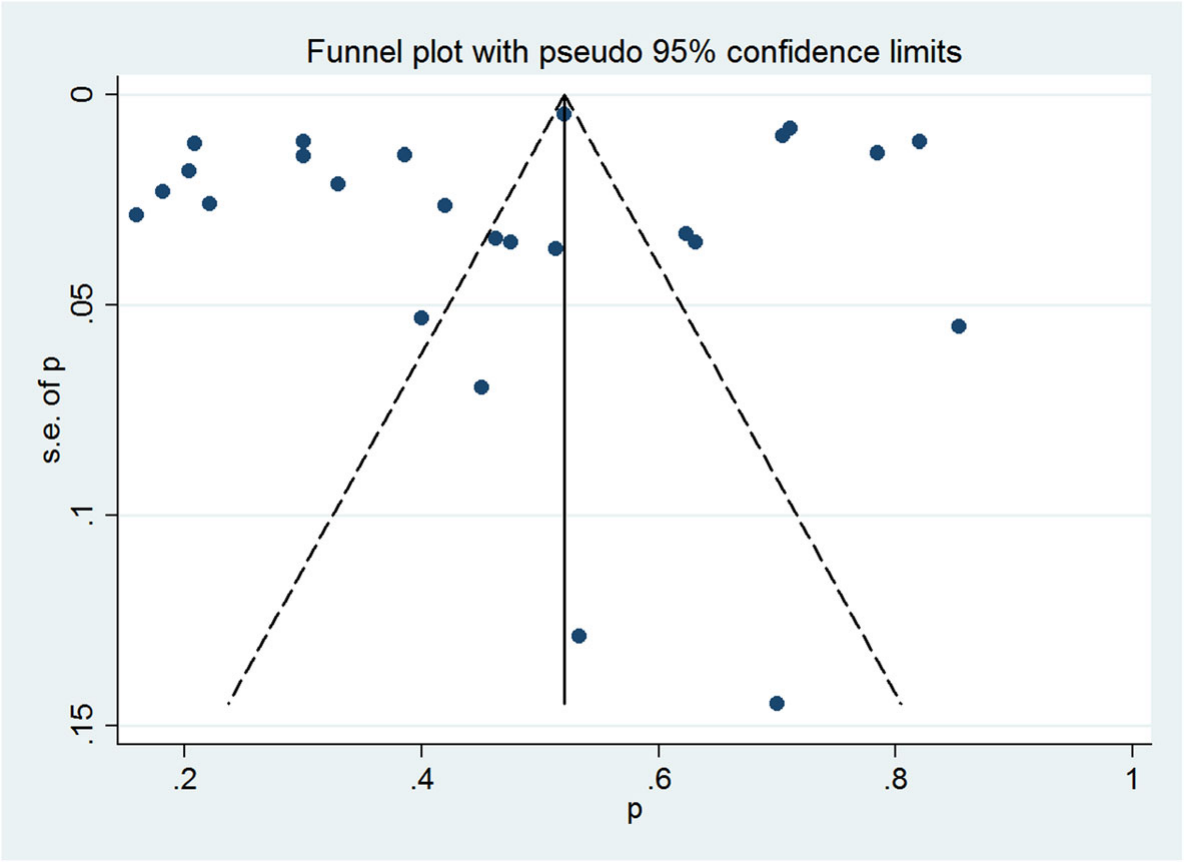
Funnel plot of the prevalence of turnover intention

A funnel plot was generated (Fig. 3) and visual inspection of it revealed no asymmetry. The Egger’s test result found no evidence of substantial publication bias (*P* = 0.25).

## Discussion

This is the first comprehensive systematic review and meta-analysis on the turnover intentions of GPs worldwide. The analysis revealed that 46% of GPs in the studies in the 25 articles published between 1988 and 2019 reported turnover intention. This comprehensive meta-analysis revealed some important results. First, turnover intention rate varied across data collection method. Second, the analysis identified factors likely associated with GPs’ turnover intentions.

Some studies investigated the GPs’ turnover intention in some regions, and the results showed that 52.7% of Australian GPs [48], 30% of New Zealand GPs [47], 23.6% of Canadian GPs [56], 52% of Finnish GPs [57], and 70.0% of Chinese GPs [30] had significant turnover intention. Our findings provide a more comprehensive reflect of GPs’ turnover intention worldwide. GPs are the main primary care providers, and governments are supposed to focus on them and implement policies to protect their rights and benefits, which might reflect a growing global emphasis on primary care.

The group comparisons regarding data collection methods revealed a higher turnover intention in the studies that combined questionnaires with interviews than those that used just questionnaire data. It was possible that GPs were more truthful in interviews than on questionnaires or that the survey questions poorly reflected the concept of turnover intention [58]. Thus, researchers should carefully assess the validity of their questions and consider ways other than surveys to obtain accurate and precise data [59].

Theory and empirical research assume that turnover intention results from an overall assessment of a job in which an employee considers a variety of extrinsic and intrinsic factors. Extrinsic factors tend to promote retention and intrinsic factors tend to increase enthusiasm and job satisfaction. Rapid social developments might have influenced job satisfaction or GPs’ incomes and standards of living in ways that influenced GPs’ turnover intention throughout the world [60]. We found that age, and work tenure were not potential predictors of turnover intention. However, limited opportunities for personal development, low salary, and a high-level professional title probably were the main extrinsic factors, which supports An et al.’s findings [61]. Limited opportunities for personal development and low salary [62, 63] may influence GPs’ incomes and studies in our sample found that financial factors were important to GPs’ turnover intention.

GPs with senior titles usually are paid high salaries, but they also have heavy workloads and high stress levels. Heavy workloads that caused stress had a strong influence on turnover intention. Heavy workloads and high stress levels reflect the changes in the labor supply of and demand for GPs around the world. Several previous studies found that workplace violence, which has been increasing on a global scale, was likely related to GPs’ turnover intention. Intrinsic factors, such as job satisfaction and morale, were probably as important to turnover intention. One interesting finding was that morale was the strongest of all the predictive factors. Many factors might contribute to low morale, and, importantly, no studies were found that examined the reasons for low morale among GPs. Researchers should investigate this important finding.

Moreover, it would be more appropriate to be skeptical and conservative on the interpretation of the predictors. These meta-analyses were based on only fewer studies and I-square of about 90%. However, the findings offer important insights for future research. These factors may affect GPs’ turnover intention to a large extent, which inspires researchers to conduct relevant large sample experiments in the future.

### Strengths and limitations

This study is the first one to investigate turnover intention and its predictors among GPs at the global level. The analyses found that extrinsic and intrinsic factors influenced the GPs’ turnover intentions. Therefore, this study is valuable to policymakers around the world who want to address employment stability in their national healthcare industries. It offers a broad perspective on the employment needs of GPs worldwide.

However, this study has some limitations to consider when interpreting and applying the findings. First, there were might be various undetected factors influencing the GPs’ turnover intentions, e.g., attitudes of spouses and family members, lack of appropriate alternative roles, and the organizational stress [64]. Second, a high heterogeneity was observed in the meta-analysis when the estimates were aggregated. This heterogeneity might relate to differences in surveymethods, sample size, medical systems and cultural backgrounds; however, the heterogeneity can be overestimated when studies with large sample sizes are pooled [65]. Third, although the definition of “turnover intention” was objective and precise, the rates might have been overestimated. GPs with higher burnout/quitting intention were more likely to response for study, as this may seem more relevant to them.

### Suggestions for future research and application

First, more studies on the influences of risk factors on turnover intention among GPs might help to reveal their fundamental concerns and provide important information that could be used to lower their turnover rates. Second, survey questionnaires should ask a greater variety of questions to gather data on additional factors, such as age, gender, and marital status. Studies focused on the causes of turnover intention among GPs would be particularly useful. Third, a definition of “turnover intention” for GPs or the healthcare industry in general should be standardized to improve comparisons across studies.

Our findings have important implications for lowering the turnover rate among GPs because they highlight the importance of preventive strategies across countries and settings. Our results strongly imply that salaries, personal development opportunities, and job satisfaction and morale are key areas to address. Raising public appreciation of GPs through mass media campaigns and protecting their employment rights and benefits might help to lower the turnover intention rate. Maintaining necessary supplies of these vital frontline providers of primary care might be supported by applying the results to appropriate policies and programs. In sum, the results of this meta-analysis revealed a moderate turnover intention rate among GPs at the global level. Prevention strategies are urgently needed to stabilize this workforce.

## Conclusions

In summary, the results of this meta-analysis indicate that the turnover intention among worldwide GPs is moderate. Prevention strategies should be developed urgently to strength the stability of GPs workforce.

## Supplementary Information

**Additional file 1: Supplementary Table 1.** Quality assessment of cross-sectional studies. **Supplementary Table 2.** Systematic review search strategy. **Supplementary Table 3.** Quality assessment of Quality in prognostic studies (QUIPS).

## Data Availability

Data may be made available by contacting the corresponding author.

## Abbreviations

GPs: General practitioners
CNKI: China National Knowledge Infrastructure
ORs: Odds ratios
CIs: Confidence intervals
