# Supplementary material for: The global prevalence of turnover intention among general practitioners: a systematic review and meta-analysis
Source: BMC Fam Pract. 2020 Nov 30;21:246. doi: 10.1186/s12875-020-01309-4 (PMC7702723; doi:10.1186/s12875-020-01309-4)
Supplement: Supplementary file 1 — Additional file 1: Supplementary Table 1. Quality assessment of cross-sectional studies. Supplementary Table 2. Systematic review search strategy. Supplementary Table 3. Quality assessment of Quality in prognostic studies (QUIPS). [file 12875_2020_1309_MOESM1_ESM.docx]

**Supplementary Table 1.Quality assessment of cross-sectional studies***

| **Author** | **Year** | **1) Define the source of information (survey, record review)** | **2) List inclusion and exclusion criteria for exposed and unexposed subjects (cases and controls) or refer to previous publications** | **3) Indicate time period used for identifying patients** | **4) Indicate whether or not subjects were consecutive if not population-based** | **5) Indicate if evaluators of subjective components of study were masked to other aspects of the status of the participants** | **6) Describe any assessments undertaken for quality assurance purposes** | **7) Explain any patient exclusions from analysis** | **8) Describe how confounding was assessed and/or controlled.** | **9) If applicable, explain how missing data were handled in the analysis** | **10) Summarize patient response rates and completeness of data collection** | **11) Clarify what follow-up, if any, was expected and the percentage of patients for which incomplete data or follow-up was obtained** | **Total quality score** |
| --- | --- | --- | --- | --- | --- | --- | --- | --- | --- | --- | --- | --- | --- |
| Smith AG et al | 1988 | 1 | 0 | 0 | 0 | 1 | 0 | 0 | 0 | 0 | 1 | 0 | 3 |
| [Montalto M et al](https://www.ncbi.nlm.nih.gov/pubmed/?term=Montalto%20M%5bAuthor%5d&cauthor=true&cauthor_uid=7718658" \o "https://www.ncbi.nlm.nih.gov/pubmed/?term=Montalto M[Author]&cauthor=true&cauthor_uid=7718658) | 1994 | 1 | 0 | 0 | 0 | 1 | 0 | 0 | 0 | 1 | 1 | 0 | 4 |
| Maria Gardiner et al | 2001 | 1 | 0 | 1 | 1 | 1 | 0 | 0 | 1 | 0 | 1 | 1 | 7 |
| Peter MacIsaac et al | 2001 | 1 | 0 | 0 | 0 | 1 | 0 | 0 | 0 | 0 | 1 | 0 | 3 |
| Catherine Joyce et al | 2003 | 1 | 0 | 0 | 0 | 1 | 0 | 0 | 1 | 0 | 1 | 0 | 4 |
| Margaret Chambers et al | 2004 | 1 | 0 | 0 | 0 | 1 | 1 | 1 | 1 | 0 | 1 | 0 | 6 |
| Maria Gardiner et al | 2005 | 1 | 0 | 0 | 0 | 1 | 1 | 0 | 1 | 0 | 1 | 0 | 5 |
| [McComb ED](https://www.ncbi.nlm.nih.gov/pubmed/?term=McComb%20ED%5bAuthor%5d&cauthor=true&cauthor_uid=18480883" \o "https://www.ncbi.nlm.nih.gov/pubmed/?term=McComb ED[Author]&cauthor=true&cauthor_uid=18480883) | 2008 | 1 | 0 | 0 | 0 | 1 | 0 | 0 | 1 | 0 | 1 | 0 | 4 |
| Tarja Heponiemi et al | 2012 | 1 | 0 | 1 | 1 | 1 | 1 | 1 | 1 | 0 | 1 | 1 | 9 |
| Yu Sun et al | 2013 | 1 | 0 | 0 | 0 | 1 | 1 | 0 | 1 | 1 | 1 | 0 | 6 |
| Maria Gardiner et al | 2013 | 1 | 1 | 0 | 0 | 1 | 0 | 0 | 1 | 1 | 1 | 1 | 7 |
| Yaming Zou et al | 2015 | 1 | 0 | 0 | 0 | 1 | 1 | 0 | 1 | 0 | 1 | 0 | 5 |
| Jeremy Dale et al | 2015 | 1 | 0 | 0 | 0 | 1 | 1 | 0 | 1 | 0 | 1 | 0 | 5 |
| Matthew et al | 2015 | 1 | 0 | 1 | 0 | 1 | 1 | 1 | 1 | 0 | 1 | 0 | 7 |
| Chang Guangming et al | 2016 | 1 | 0 | 0 | 0 | 1 | 1 | 1 | 1 | 1 | 1 | 0 | 7 |
| YU Yongli et al | 2016 | 1 | 0 | 0 | 0 | 1 | 1 | 0 | 1 | 0 | 1 | 0 | 5 |
| Emily Fletcher et al | 2016 | 1 | 0 | 0 | 0 | 1 | 1 | 1 | 1 | 1 | 1 | 0 | 7 |
| Gareth Iacobucci et al | 2016 | 1 | 0 | 0 | 0 | 1 | 0 | 0 | 0 | 0 | 1 | 0 | 3 |
| Mari et al | 2017 | 1 | 0 | 0 | 0 | 1 | 0 | 1 | 1 | 1 | 1 | 0 | 6 |
| Chen Dingwan et al | 2017 | 1 | 0 | 0 | 0 | 1 | 1 | 1 | 1 | 0 | 1 | 0 | 6 |
| FAN En-fang et al | 2017 | 1 | 0 | 0 | 0 | 1 | 1 | 0 | 1 | 0 | 1 | 0 | 5 |
| Yong Gan et al | 2018 | 1 | 0 | 0 | 0 | 1 | 1 | 1 | 1 | 1 | 1 | 0 | 7 |
| Anna Sansom et al | 2018 | 1 | 0 | 0 | 0 | 1 | 1 | 0 | 1 | 1 | 1 | 0 | 6 |
| Ouweilin et al | 2018 | 1 | 0 | 0 | 0 | 1 | 1 | 0 | 1 | 0 | 1 | 0 | 5 |
| [Yong Gan et al](https://onlinelibrary.wiley.com/action/doSearch?ContribAuthorStored=Gan,+Yong) | 2019 | 1 | 0 | 0 | 0 | 1 | 1 | 1 | 1 | 1 | 1 | 0 | 7 |
| *The study quality was assessed according to the 11 items recommended by the Agency for Healthcare Research and Quality (AHRQ) for cross-sectional studies. 1 point if the item was contemplated in the study, 0 point if the item was not, and unable to determine. 1 = “Yes”, 0 = “No”, “Unable to determine”, or “Not applicable” | | | | | | | | | | | | | |

**Supplementary Table2: Systematic review search strategy**

| 1.'general practitioners'. |
| --- |
| 2.'GPs'. |
| 3.'health worker'. |
| 4.1 or 2 or 3 |
| 5.'turnover'. |
| 6.'turnover intention'. |
| 7.'demission'. |
| 8.'retain'. |
| 9.5 or 6 or 7 or 8 |

**Supplementary Table 3. Quality assessment of Quality in prognostic studies (QUIPS)**

| **Study** | **Study participation (max. 15)** | **Study attrition (max. 15)** | **Prognostic factor measurement (max. 15)** | **Confounding measurement and account (max. 15)** | **Outcome measurement (max. 15)** | **Statistical analysis and reporting (max. 15)** | **Quality score (max. 90)** |
| --- | --- | --- | --- | --- | --- | --- | --- |
| Smith et al (1988) | 10 | 5 | 10 | 12.5 | 10 | 12.5 | 60 |
| [Montalto et al (1994)](https://www.ncbi.nlm.nih.gov/pubmed/?term=Montalto M[Author]&cauthor=true&cauthor_uid=7718658" \o "https://www.ncbi.nlm.nih.gov/pubmed/?term=Montalto M[Author]&cauthor=true&cauthor_uid=7718658) | 10 | 5 | 10 | 15 | 7.5 | 12.5 | 60 |
| Gardiner et al (2001) | 12.5 | 5 | 10 | 15 | 10 | 15 | 67.5 |
| MacIsaac et al (2001) | 15 | 5 | 10 | 12.5 | 10 | 15 | 67.5 |
| Joyce et al (2003) | 15 | 5 | 10 | 12.5 | 7.5 | 15 | 65 |
| Chambers et al (2004) | 12.5 | 5 | 10 | 15 | 7.5 | 12.5 | 62.5 |
| Gardiner et al (2005) | 15 | 5 | 10 | 15 | 10 | 15 | 70 |
| [Comb ED (2008)](https://www.ncbi.nlm.nih.gov/pubmed/?term=McComb ED[Author]&cauthor=true&cauthor_uid=18480883" \o "https://www.ncbi.nlm.nih.gov/pubmed/?term=McComb ED[Author]&cauthor=true&cauthor_uid=18480883) | 15 | 5 | 10 | 12.5 | 12.5 | 15 | 70 |
| Heponiemi et al (2012) | 15 | 5 | 10 | 12.5 | 10 | 15 | 67.5 |
| Sun et al (2013) | 10 | 5 | 12.5 | 15 | 7.5 | 15 | 65 |
| Gardiner et al (2013) | 15 | 5 | 12.5 | 15 | 10 | 12.5 | 70 |
| Zou et al (2015) | 15 | 5 | 12.5 | 15 | 12.5 | 15 | 75 |
| Dale et al (2015) | 15 | 5 | 10 | 12.5 | 12.5 | 15 | 70 |
| Matthew et al (2015) | 10 | 5 | 12.5 | 15 | 12.5 | 15 | 70 |
| Chang et al (2016) | 15 | 5 | 12.5 | 15 | 12.5 | 12.5 | 72.5 |
| Yu et al (2016) | 12.5 | 5 | 10 | 15 | 12.5 | 15 | 70 |
| Fletcher et al (2016) | 15 | 5 | 12.5 | 15 | 12.5 | 15 | 75 |
| Iacobucci et al (2016) | 15 | 5 | 12.5 | 12.5 | 12.5 | 12.5 | 70 |
| Mari et al (2017) | 10 | 5 | 10 | 15 | 12.5 | 15 | 67.5 |
| Chen et al (2017) | 15 | 5 | 12.5 | 15 | 15 | 15 | 77.5 |
| Fan et al (2017) | 15 | 5 | 12.5 | 12.5 | 10 | 12.5 | 67.5 |
| Gan et al (2018) | 15 | 5 | 10 | 15 | 7.5 | 15 | 67.5 |
| Sansom et al (2018) | 12.5 | 5 | 12.5 | 15 | 15 | 15 | 75 |
| Ouweilin et al (2018) | 15 | 5 | 12.5 | 15 | 10 | 15 | 72.5 |
| [Gan et al (2018)](https://onlinelibrary.wiley.com/action/doSearch?ContribAuthorStored=Gan,+Yong) | 15 | 5 | 12.5 | 15 | 12.5 | 15 | 75 |
